# (5-Fluoro-2,6-dioxo-1,2,3,6-tetra­hydro­pyrimidin-1-ido-κ*N* ^1^)(1,4,8,11-tetra­aza­cyclo­tetra­decane-κ^4^ *N*)zinc(II) perchlorate

**DOI:** 10.1107/S2414314624004310

**Published:** 2024-05-21

**Authors:** Yoshimi Ichimaru, Koichi Kato, Wanchun Jin, Masaaki Kurihara, Hiromasa Kurosaki

**Affiliations:** a Faculty of Pharmaceutical Sciences, Shonan University of Medical Sciences, 16-48, Kamishinano, Totsuka-ku, Yokohama, Kanagawa 244-0806, Japan; b College of Pharmacy, Kinjo Gakuin University, 2-1723 Omori, Nagoya 463-8521, Japan; Goethe-Universität Frankfurt, Germany

**Keywords:** crystal structure, zinc(II) complex, cyclam, [14]aneN4, fluoro­uracil

## Abstract

In the structure of the title complex, [Zn(C_4_H_2_FN_2_O_2_)(C_10_H_24_N_4_)]ClO_4_, the zinc(II) ion forms coordination bonds with the four nitro­gen atoms of cyclam (1,4,8,11-tetra­aza­cyclo­tetra­decane or [14]aneN4) as well as with the nitro­gen atom of a deprotonated 5-fluoro­uracil ion (FU^−^). Cyclam adopts a *trans*-I type conformation within this structure. The coordination structure of the zinc(II) ion is a square pyramid with a distorted base plane formed by the four nitro­gen atoms of the cyclam. FU^−^ engages in inter­molecular hydrogen bonding with neighboring FU^−^ mol­ecules and with the cyclam mol­ecule.

## Abstract 2

In the structure of the title complex, the zinc(II) ion forms coordination bonds with the four nitro­gen atoms of cyclam as well as with the nitro­gen atom of a deprotonated 5-fluoro­uracil ion. Cyclam adopts a *trans*-I type conformation within this structure. The coordination structure of the zinc(II) ion is a square pyramid with a distorted base plane formed by the four nitro­gen atoms of the cyclam.

## Graphical abstract

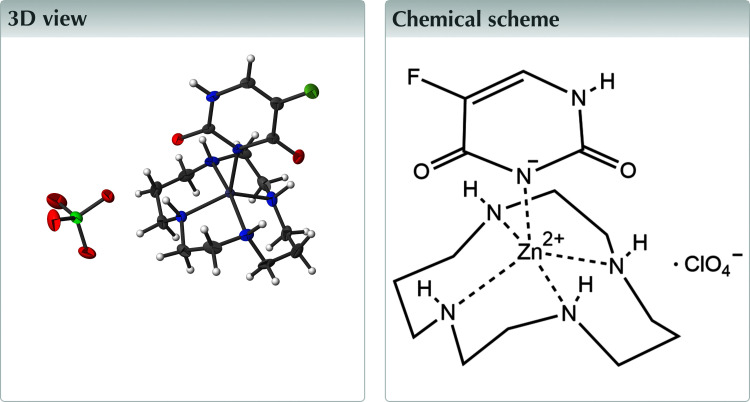

## Structure description

Cyclam (= 1,4,8,11-tetra­aza­cyclo­tetra­decane or [14]aneN4) is a widely recognized macrocyclic polyamine renowned for its strong chelation properties with transition-metal cations, such as cobalt(III) ion (Fang *et al.*, 2024 ▸), copper(II) ion (Emsley *et al.*, 1990 ▸), and nickel(II) ion (Prasad *et al.*, 1987 ▸). We have reported the crystal structure of a zinc(II) ion and a cyclam complex (Zn^II^–cyclam) (Ichimaru *et al.*, 2022 ▸). Cyclen (= 1,4,7,10-tetra­aza­cyclo­dodecane or [12]aneN4) shares similarities with cyclam as a macrocyclic polyamine. Cyclen’s chelation properties with metal cations are largely akin to those of cyclam, including its affinity for zinc(II) ions (Ichimaru *et al.*, 2021 ▸). Cyclam and cyclen differ in the number of atoms forming their rings. In metal–cyclam complexes, the metal cation and four nitro­gen atoms lie within the same plane, allowing for the coordination of two counter-anions perpendicular to the plane; the coordination structure of a central metal is octa­hedral. Conversely, in metal–cyclen complexes, the metal cation is located above the plane formed by the four nitro­gen atoms, enabling the coordination of one counter anion; the coordination structure of a central metal is a square pyramid. We previously reported on the formation of a complex between deprotonated 5-fluoro­uracil (FU^−^) and Zn^II^–cyclen (Ichimaru *et al.*, 2023 ▸). In this study, we attempted to synthesize the aforementioned complex by reacting Zn^II^–cyclam with FU^−^ at a 1:2 stoichiometry. However, the resulting mol­ecule was identified as the title complex, that is, Zn^II^–cyclam and FU^−^ formed a complex with 1:1 stoichiometry. Further studies on the reaction conditions for complex formation, including changing the reaction stoichiometry, are expected in the future.

**Figure 1:**
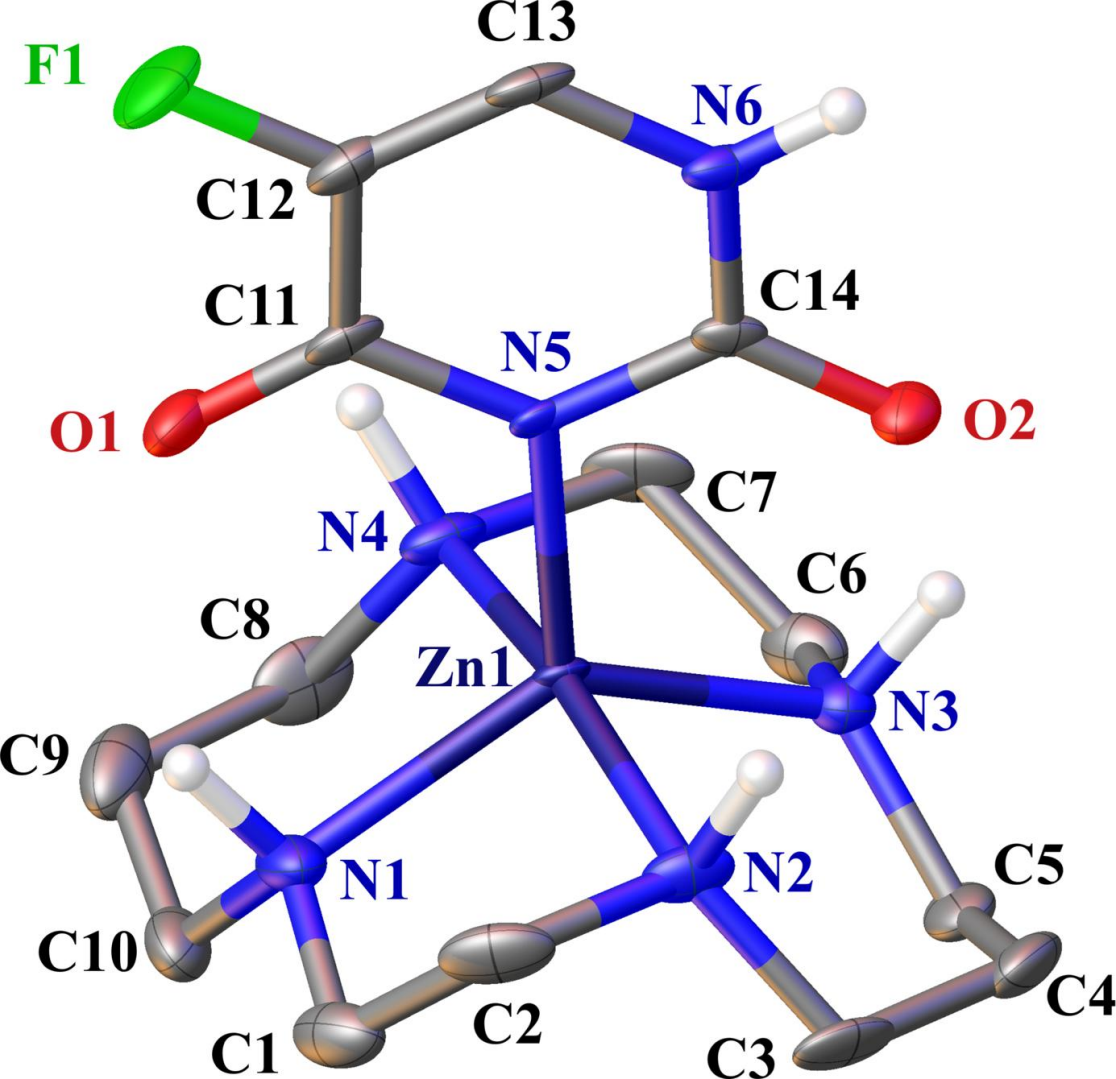
The title complex with displacement ellipsoids drawn at the 50% probability level. C-bound H atoms and a perchlorate ion are omitted for clarity.

**Figure 2:**
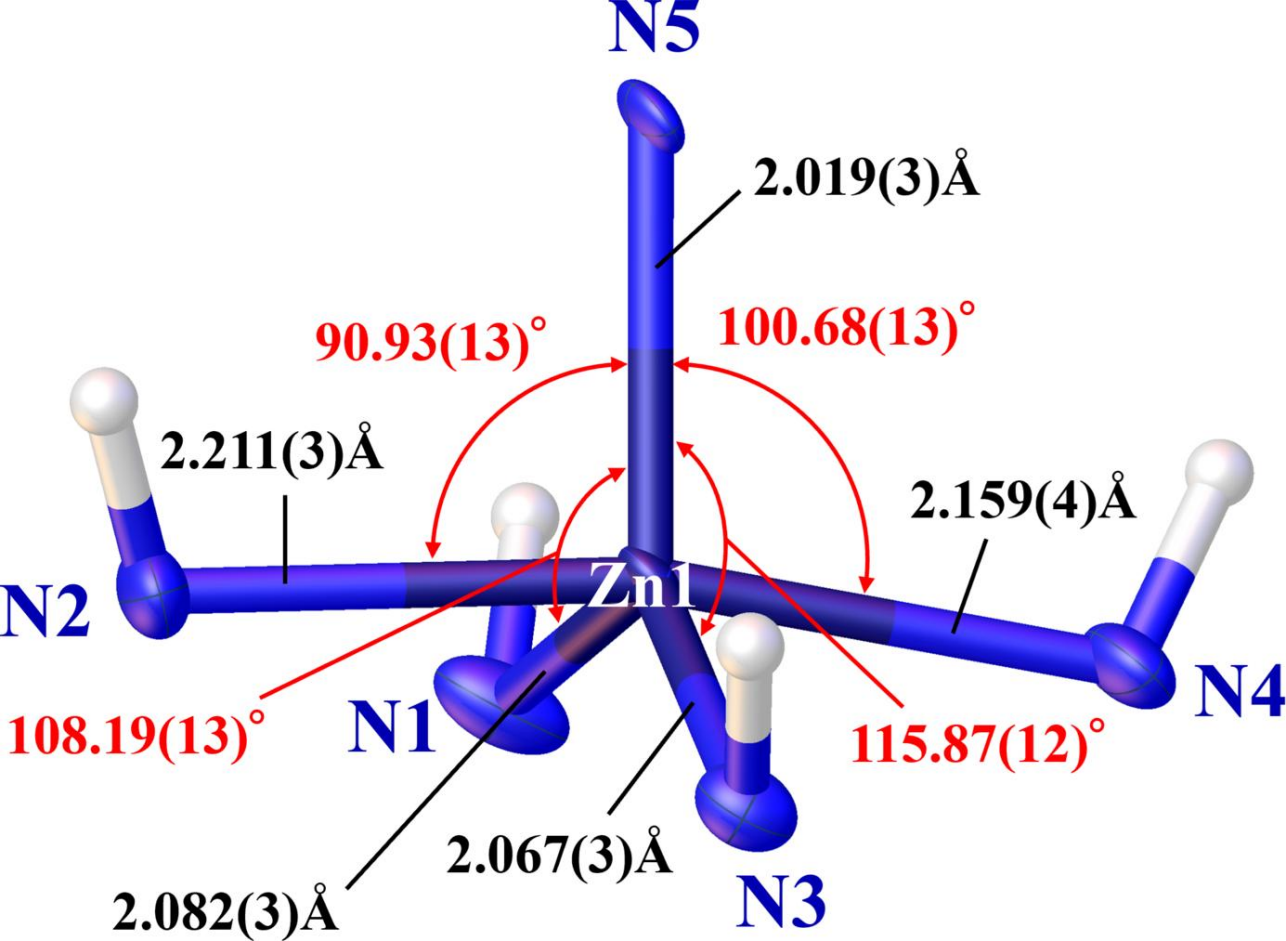
The coordination structure of Zn1 showing with displacement ellipsoids drawn at the 50% probability level. Bond angles and bond lengths are shown in red and black, respectively.

The title complex comprises an FU^−^ bound to a zinc(II) ion chelated by cyclam (Fig. 1 ▸). The FU^−^ mol­ecule was formed by deprotonation of the N–H group at the most acidic 3-position of FU. Additionally, one perchlorate ion serves as a counter-anion adjacent to the complex. In terms of the cyclam ring’s conformation within the title complex, it adopts a *trans*-I (*R*, *S*, *R*, *S*) type, while the energetically most stable coordination is the *trans*-III (*R*, *R*, *S*, *S*) type (Bosnich *et al.*, 1965 ▸; Oakley *et al.*, 2024 ▸). In instances where the central metal is a zinc(II) ion, it is noted that two counter-anions can coordinate perpendicular to the plane established by the *trans*-III type cyclam (Ichimaru *et al.*, 2022 ▸). However, in the title complex, contrary to our expectation, only one FU^−^ mol­ecule coordinates with the zinc(II) ion, while the cyclam adopts a *trans*-I type. In cases where an anion coordinates with the central metal of *N*-tetra­methyl­cyclam, the *trans*-I type is often adopted, primarily due to non-bonding inter­actions (Liang & Sadler, 2004 ▸). However, it is uncommon for *N*-non-substituted cyclam to adopt the *trans*-I type. The coordination system of zinc(II) ion is shown in Fig. 2 ▸. The bond angles formed by N5—Zn1 and the nitro­gen atoms of cyclam (N1, N2, N3, and N4) are 108.17 (13), 90.91 (13), 115.88 (13), and 100.68 (13)°, respectively. These bond angles were observed to be smaller for the longer bond lengths between Zn1 and the nitro­gen atoms of cyclam (N1, N2, N3, and N4). In a typical Zn^II^–cyclam complex, the corresponding bond angles and bond lengths are approximately 90° and 2.08 Å, respectively (Ichimaru *et al.*, 2022 ▸). In the title compound, the bond Z1—N2 is 2.211 (3) Å when the bond angle N5—Zn1—N2 is 90.91 (13)°, and the distance Zn1—N1 equals 2.082 (3) Å when the bond angle N5—Zn1—N1 amounts to 108.17 (13)°. The distances between two pairs of N atoms located diagonally across Zn1 are N1⋯N3 = 3.840 (5) and N2⋯N4 = 4.349 (5) Å. In a typical Zn^II^–cyclam complex, the corresponding distance is approximately 4.17 Å (Ichimaru *et al.*, 2022 ▸). As discussed earlier, the nitro­gen atoms of the cyclam are not situated in the same plane. The coordination structure of the zinc(II) ion is a distorted square pyramid. The zinc(II) ion is located 0.5034 (18) Å vertically above the centroid of the mean plane formed by the nitro­gen atoms of the cyclam.

**Figure 3:**
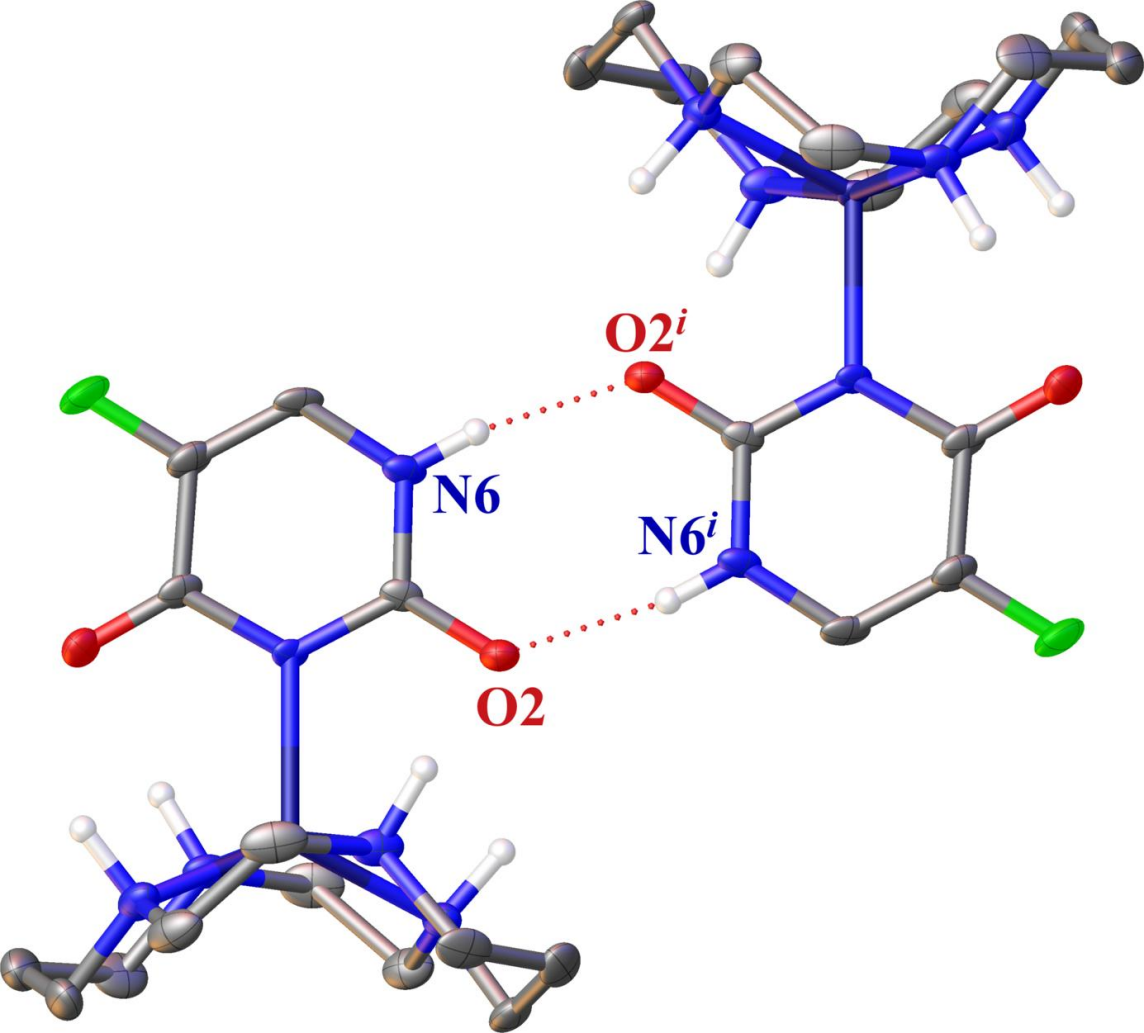
The inter­molecular hydrogen-bonding inter­actions of the title complex with displacement ellipsoids drawn at the 50% probability level. C-bound H atoms are omitted for clarity. Hydrogen-bonding inter­actions are shown as dotted lines. [Symmetry code: (*i*) 1 − *x*, 1 − *y*, 1 − *z*.]

**Figure 4:**
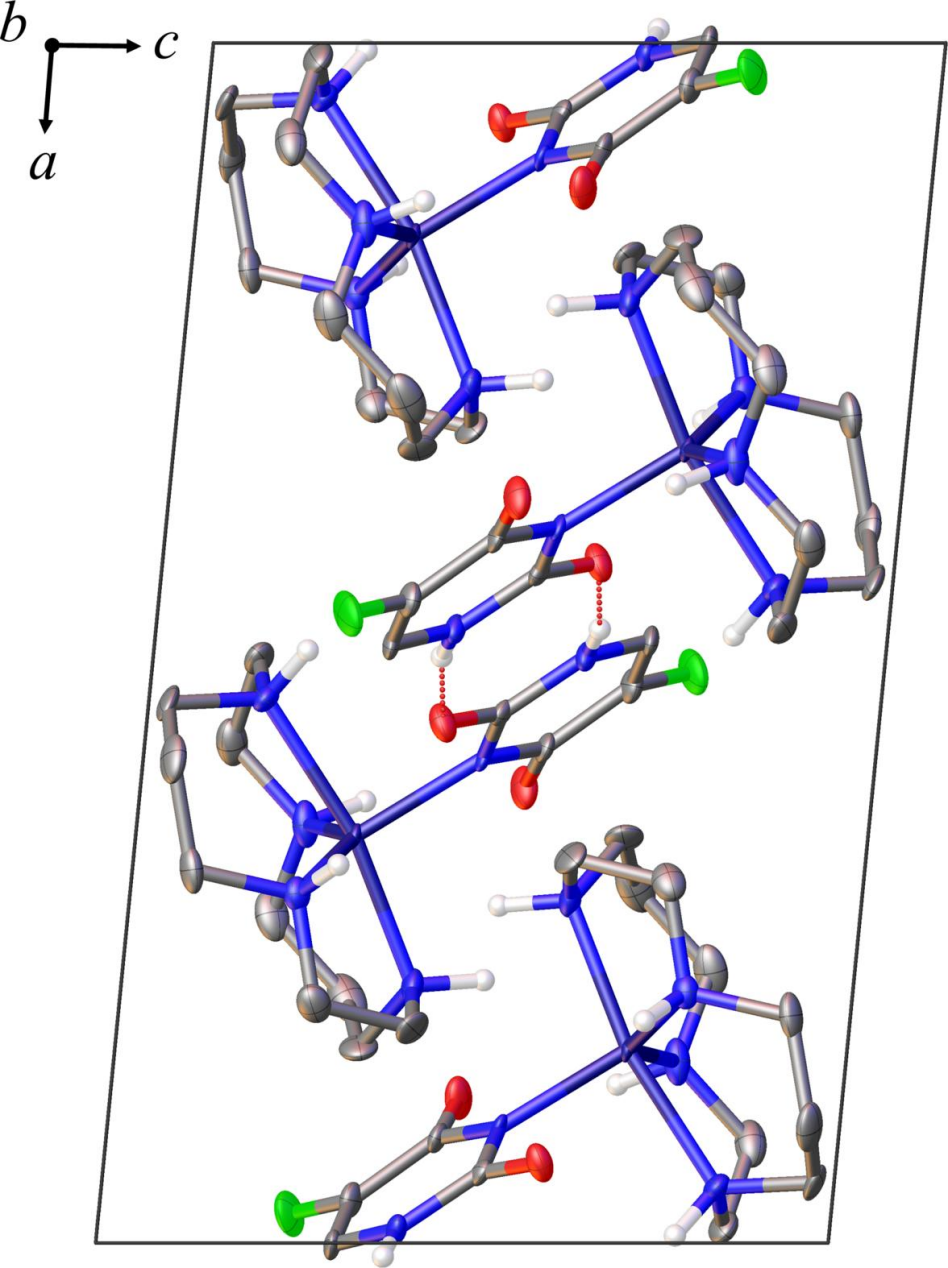
Packing view down *b*-axis of the title complex showing with displacement ellipsoids drawn at the 50% probability level. Perchlorate ions and C-bound H atoms are omitted for clarity. Hydrogen-bonding inter­actions are shown as dotted lines.

FU^−^ mol­ecules engage in inter­molecular hydrogen bonding with neighboring FU^−^ mol­ecules (Fig. 3 ▸). In addition, there are intra­molecular hydrogen bonds between the carbonyl groups and the NH moieties of the cyclam. The perchlorate ion forms hydrogen bonds to two different cyclam rings. Table 1 ▸ provides a summary of numerical data related to hydrogen bonding. The hydrogen bonding involves the N6—H6 group, which is not coordinated with the cyclam, and the oxygen atom (O2) of the carbonyl group adjacent to the N6—H6 group. Similar hydrogen-bond formations are observed in complexes of FU^−^ and zinc(II) ions other than the title complex (Icsel *et al.*, 2022 ▸). Even in crystals where a complex has not formed, two FU mol­ecules form a hydrogen bond similar to that in the title compound (Hulme & Tocher, 2004 ▸). However, the N—H group participating in this hydrogen bonding is different from that of the title compound, that is, a more acidic N—H is involved in the hydrogen bond. In the title complex, the highly acidic hydrogen atom is released, allowing another N—H to form a hydrogen bond. In polyamine complexes such as Zn^II^–cyclen and Zn^II^–cyclam, the N—H group involved in ring formation can contribute to the hydrogen bonding network with counter anions and/or ligands (Ichimaru *et al.*, 2021 ▸; Donaghy *et al.*, 2023 ▸). In our previously reported complex of FU^−^ bound to Zn^II^–cyclen, the carbonyl oxygen of FU^−^ formed hydrogen bonds with the N—H of cyclen and a perchlorate ion (Ichimaru *et al.*, 2023 ▸). The torsion angles between the two carbonyl groups of FU^−^ and the two pairs of nitro­gen atoms (N1 and N3, N2, and N4) located diagonally across the zinc(II) ion are O1—O2—N3—N1 = −25.84 (8)° and O2—O1—N2—N4 = −76.57 (11)°. This indicates that the two carbonyl groups are not aligned parallel to either of the two pairs of nitro­gen atoms situated at opposite angles. A packing diagram is provided in Fig. 4 ▸. Besides the aforementioned hydrogen bonding, no other inter­molecular inter­actions were observed.

## Synthesis and crystallization

[Zn^II^–cyclam](ClO_4_)_2_ was synthesized using a previously reported method (Tyson *et al.*, 1990 ▸). 5-Fluoro­uracil (60.0 mg, 0.46 mmol) in 4.54 ml of H_2_O, 0.46 ml of 1 mol L^−1^ NaOH aq was added to clarify the suspension. After stirring at room temperature for 30 min, a solution of aqueous [Zn^II^–cyclam](ClO_4_)_2_ (107.1 mg, 0.23 mmol, 2.0 ml) was added dropwise to the reaction mixture; it was then stirred at 323 K for 4 h. Subsequently, the reaction mixture was filtered through a cellulose acetate filter (0.22-µm pore size) and then allowed to stand overnight at room temperature. Colorless crystals suitable for X-ray crystallographic analysis were obtained.

## Refinement

Crystal data, data collection and structure refinement details are summarized in Table 2 ▸.

## Supplementary Material

Crystal structure: contains datablock(s) I. DOI: 10.1107/S2414314624004310/bt4149sup1.cif

Structure factors: contains datablock(s) I. DOI: 10.1107/S2414314624004310/bt4149Isup2.hkl

CCDC reference: 2354211

Additional supporting information:  crystallographic information; 3D view; checkCIF report

## Figures and Tables

**Table 1:** Hydrogen-bond geometry (Å, °)

*D*—H⋯*A*	*D*—H	H⋯*A*	*D*⋯*A*	*D*—H⋯*A*
N6—H6⋯O2^i^	0.88	1.94	2.803 (4)	167
N1—H1⋯O1	1.00	2.19	2.966 (4)	133
N3—H3⋯O2	1.00	2.37	3.066 (4)	126
N3—H3⋯O5	1.00	2.24	3.055 (4)	138
N4—H4⋯O5^ii^	1.00	2.44	3.403 (5)	161
N4—H4⋯O6^ii^	1.00	2.44	3.229 (5)	136

Symmetry codes: (i) 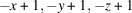 ; (ii) 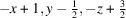 .

**Table 2:** Experimental details

Crystal data	
Chemical formula	[Zn(C_4_H_2_FN_2_O_2_)(C_10_H_24_N_4_)]ClO_4_
*M*_r_	494.23
Crystal system, space group	Monoclinic, *P*2_1_/*c*
Temperature (K)	100
*a*, *b*, *c* (Å)	9.8065 (2), 12.5029 (3), 16.1592 (3)
β (°)	95.599 (2)
*V* (Å^3^)	1971.82 (7)
*Z*	4
Radiation type	Cu *K*α
μ (mm^−1^)	3.48
Crystal size (mm)	0.91 × 0.64 × 0.55

Data collection	
Diffractometer	Rigaku XtaLAB Synergy-i
Absorption correction	Multi-scan (*CrysAlis PRO*; Rigaku OD, 2022 ▸)
*T*_min_, *T*_max_	0.465, 1.000
No. of measured, independent and observed [*I* > 2σ(*I*)] reflections	17193, 3587, 3489
*R*_int_	0.092
(sin θ/λ)_max_ (Å^−1^)	0.602

Refinement	
*R*[*F* ^2^ > 2σ(*F* ^2^)], *wR*(*F* ^2^), *S*	0.064, 0.179, 1.11
No. of reflections	3587
No. of parameters	262
H-atom treatment	H-atom parameters constrained
Δρ_max_, Δρ_min_ (e Å^−3^)	1.60, −1.36

Computer programs: *CrysAlis PRO* (Rigaku OD, 2022 ▸), *SHELXT2018/2* (Sheldrick, 2015*a* ▸), *SHELXL2019/3* (Sheldrick, 2015*b* ▸) and *OLEX2* (Dolomanov *et al.*, 2009 ▸).

## full crystallographic data

### Crystal data

**Table:**

[Zn(C_4_H_2_FN_2_O_2_)(C_10_H_24_N_4_)]ClO_4_	*F*(000) = 1024
*M**_r_* = 494.23	*D*_x_ = 1.665 Mg m^−^^3^
Monoclinic, *P*2_1_/*c*	Cu *K*α radiation, λ = 1.54184 Å
*a* = 9.8065 (2) Å	Cell parameters from 13648 reflections
*b* = 12.5029 (3) Å	θ = 2.7–68.1°
*c* = 16.1592 (3) Å	µ = 3.48 mm^−^^1^
β = 95.599 (2)°	*T* = 100 K
*V* = 1971.82 (7) Å^3^	Block, colourless
*Z* = 4	0.91 × 0.64 × 0.55 mm

### Data collection

**Table:**

Rigaku XtaLAB Synergy-i diffractometer	3587 independent reflections
Radiation source: microfocus sealed X-ray tube, PhotonJet-i	3489 reflections with *I* > 2σ(*I*)
Multi-layer mirror optics monochromator	*R*_int_ = 0.092
Detector resolution: 10.0 pixels mm^-1^	θ_max_ = 68.2°, θ_min_ = 4.5°
ω scans	*h* = −11→11
Absorption correction: multi-scan (CrysAlisPro; Rigaku OD, 2022)	*k* = −14→14
*T*_min_ = 0.465, *T*_max_ = 1.000	*l* = −19→19
17193 measured reflections	

### Refinement

**Table:**

Refinement on *F*^2^	Primary atom site location: dual
Least-squares matrix: full	Hydrogen site location: inferred from neighbouring sites
*R*[*F*^2^ > 2σ(*F*^2^)] = 0.064	H-atom parameters constrained
*wR*(*F*^2^) = 0.179	*w* = 1/[σ^2^(*F*_o_^2^) + (0.1099*P*)^2^ + 4.5604*P*] where *P* = (*F*_o_^2^ + 2*F*_c_^2^)/3
*S* = 1.11	(Δ/σ)_max_ = 0.002
3587 reflections	Δρ_max_ = 1.60 e Å^−^^3^
262 parameters	Δρ_min_ = −1.36 e Å^−^^3^
0 restraints	

### Special details

**Table:**

Geometry. All esds (except the esd in the dihedral angle between two l.s. planes) are estimated using the full covariance matrix. The cell esds are taken into account individually in the estimation of esds in distances, angles and torsion angles; correlations between esds in cell parameters are only used when they are defined by crystal symmetry. An approximate (isotropic) treatment of cell esds is used for estimating esds involving l.s. planes.
Refinement. All hydrogen atoms were located by a geometrical calculation, and were not refined.

### Fractional atomic coordinates and isotropic or equivalent isotropic displacement parameters (Å^2^)

**Table:**

	*x*	*y*	*z*	*U*_iso_*/*U*_eq_	
Zn1	0.29929 (5)	0.24605 (3)	0.66114 (3)	0.0114 (2)	
Cl1	0.20118 (9)	0.70678 (7)	0.71977 (6)	0.0210 (3)	
F1	0.7396 (2)	0.1048 (2)	0.52427 (16)	0.0335 (6)	
O2	0.4045 (3)	0.4332 (2)	0.56678 (17)	0.0222 (6)	
O1	0.5241 (3)	0.0863 (2)	0.62077 (19)	0.0260 (6)	
N5	0.4640 (3)	0.2604 (2)	0.5961 (2)	0.0137 (7)	
O5	0.2791 (3)	0.6245 (2)	0.6828 (2)	0.0318 (7)	
N6	0.5912 (3)	0.3668 (3)	0.51134 (19)	0.0185 (7)	
H6	0.606066	0.429211	0.488664	0.022*	
O3	0.1333 (4)	0.6626 (3)	0.7866 (2)	0.0404 (8)	
N3	0.2281 (3)	0.3864 (2)	0.7091 (2)	0.0188 (7)	
H3	0.280721	0.445380	0.685020	0.023*	
N2	0.1593 (4)	0.2636 (3)	0.5461 (2)	0.0225 (8)	
H2	0.210131	0.305494	0.506244	0.027*	
N4	0.3982 (3)	0.2264 (3)	0.7856 (2)	0.0226 (7)	
H4	0.494546	0.202495	0.781057	0.027*	
O6	0.2940 (3)	0.7882 (2)	0.7519 (2)	0.0373 (8)	
O4	0.1026 (4)	0.7510 (3)	0.6582 (3)	0.0483 (11)	
N1	0.2305 (3)	0.0887 (3)	0.6509 (2)	0.0257 (8)	
H1	0.308861	0.047100	0.631959	0.031*	
C11	0.5473 (4)	0.1728 (3)	0.5874 (2)	0.0185 (8)	
C14	0.4839 (4)	0.3561 (3)	0.5584 (2)	0.0160 (7)	
C12	0.6572 (4)	0.1887 (3)	0.5358 (3)	0.0226 (8)	
C13	0.6767 (4)	0.2824 (4)	0.4984 (2)	0.0206 (8)	
H13	0.748516	0.290368	0.463452	0.025*	
C6	0.2659 (4)	0.3903 (3)	0.8005 (3)	0.0266 (9)	
H6A	0.194817	0.353807	0.829715	0.032*	
H6B	0.271740	0.465610	0.819480	0.032*	
C7	0.4033 (4)	0.3354 (4)	0.8208 (3)	0.0294 (10)	
H7A	0.476186	0.377144	0.797392	0.035*	
H7B	0.425105	0.331652	0.881891	0.035*	
C3	0.0264 (4)	0.3177 (4)	0.5502 (3)	0.0283 (10)	
H3A	−0.035405	0.270211	0.578256	0.034*	
H3B	−0.016100	0.330925	0.492978	0.034*	
C5	0.0810 (4)	0.4135 (3)	0.6891 (3)	0.0261 (9)	
H5A	0.061496	0.482004	0.716349	0.031*	
H5B	0.024268	0.357396	0.712116	0.031*	
C2	0.1450 (4)	0.1550 (4)	0.5130 (3)	0.0311 (10)	
H2A	0.229626	0.133955	0.488375	0.037*	
H2B	0.067678	0.151948	0.468791	0.037*	
C8	0.3328 (5)	0.1478 (4)	0.8374 (3)	0.0315 (10)	
H8A	0.243074	0.176396	0.850267	0.038*	
H8B	0.390688	0.138891	0.890634	0.038*	
C1	0.1190 (4)	0.0788 (4)	0.5827 (3)	0.0310 (10)	
H1A	0.030078	0.095803	0.603804	0.037*	
H1B	0.114871	0.004402	0.561604	0.037*	
C4	0.0412 (4)	0.4232 (4)	0.5966 (3)	0.0313 (10)	
H4A	0.111204	0.466879	0.572068	0.038*	
H4B	−0.046910	0.462195	0.587902	0.038*	
C10	0.1970 (5)	0.0352 (3)	0.7273 (3)	0.0311 (10)	
H10A	0.174303	−0.040501	0.714531	0.037*	
H10B	0.114558	0.069387	0.746486	0.037*	
C9	0.3108 (5)	0.0393 (4)	0.7964 (3)	0.0370 (11)	
H9A	0.396983	0.017282	0.774066	0.044*	
H9B	0.291247	−0.013409	0.839419	0.044*	

### Atomic displacement parameters (Å^2^)

**Table:**

	*U*^11^	*U*^22^	*U*^33^	*U*^12^	*U*^13^	*U*^23^
Zn1	0.0045 (3)	0.0164 (3)	0.0130 (3)	−0.00001 (15)	−0.0012 (2)	0.00062 (16)
Cl1	0.0161 (5)	0.0239 (5)	0.0228 (5)	−0.0025 (3)	0.0017 (4)	−0.0047 (3)
F1	0.0190 (12)	0.0423 (15)	0.0409 (15)	0.0160 (11)	0.0114 (11)	0.0067 (12)
O2	0.0159 (13)	0.0226 (13)	0.0293 (15)	0.0014 (11)	0.0087 (11)	0.0055 (11)
O1	0.0149 (13)	0.0264 (14)	0.0370 (16)	0.0072 (11)	0.0043 (12)	0.0098 (12)
N5	0.0052 (14)	0.0212 (16)	0.0141 (16)	0.0008 (11)	−0.0025 (12)	0.0044 (11)
O5	0.0373 (17)	0.0244 (15)	0.0364 (17)	0.0027 (13)	0.0177 (14)	−0.0023 (13)
N6	0.0085 (14)	0.0293 (17)	0.0175 (16)	−0.0018 (12)	−0.0002 (12)	0.0070 (13)
O3	0.046 (2)	0.0431 (19)	0.0362 (18)	−0.0070 (16)	0.0235 (16)	−0.0082 (15)
N3	0.0142 (15)	0.0187 (15)	0.0235 (17)	−0.0018 (12)	0.0027 (12)	0.0001 (13)
N2	0.0134 (17)	0.039 (2)	0.0148 (17)	−0.0011 (14)	−0.0029 (13)	0.0009 (14)
N4	0.0096 (16)	0.0353 (18)	0.0227 (18)	0.0010 (14)	0.0001 (13)	0.0054 (15)
O6	0.0218 (16)	0.0215 (15)	0.067 (2)	−0.0057 (13)	−0.0039 (15)	−0.0077 (15)
O4	0.035 (2)	0.077 (3)	0.032 (2)	0.0136 (17)	−0.0048 (17)	−0.0084 (16)
N1	0.0142 (16)	0.0202 (16)	0.044 (2)	−0.0012 (13)	0.0062 (14)	−0.0012 (15)
C11	0.0074 (16)	0.030 (2)	0.0176 (18)	0.0036 (15)	−0.0030 (14)	0.0009 (15)
C14	0.0086 (16)	0.0249 (18)	0.0137 (17)	−0.0042 (14)	−0.0033 (13)	0.0017 (14)
C12	0.0103 (17)	0.032 (2)	0.025 (2)	0.0058 (16)	−0.0006 (15)	0.0011 (17)
C13	0.0057 (17)	0.038 (2)	0.0181 (19)	0.0009 (16)	−0.0005 (14)	0.0044 (17)
C6	0.024 (2)	0.027 (2)	0.029 (2)	−0.0044 (17)	0.0063 (17)	−0.0105 (17)
C7	0.019 (2)	0.048 (3)	0.020 (2)	−0.0122 (19)	−0.0057 (16)	−0.0106 (19)
C3	0.0088 (17)	0.050 (3)	0.025 (2)	0.0003 (18)	−0.0067 (15)	0.0113 (19)
C5	0.0123 (18)	0.0234 (19)	0.044 (2)	0.0036 (15)	0.0084 (17)	−0.0005 (18)
C2	0.0163 (19)	0.055 (3)	0.021 (2)	−0.0066 (19)	−0.0030 (16)	−0.012 (2)
C8	0.029 (2)	0.052 (3)	0.0137 (19)	0.005 (2)	−0.0008 (16)	0.0146 (19)
C1	0.020 (2)	0.030 (2)	0.044 (3)	−0.0044 (17)	0.0064 (18)	−0.0156 (19)
C4	0.0132 (19)	0.032 (2)	0.048 (3)	0.0081 (17)	0.0009 (18)	0.013 (2)
C10	0.025 (2)	0.0191 (19)	0.051 (3)	−0.0006 (16)	0.012 (2)	0.0098 (18)
C9	0.034 (2)	0.032 (2)	0.049 (3)	0.0103 (19)	0.018 (2)	0.020 (2)

### Geometric parameters (Å, º)

**Table:**

Zn1—N5	2.019 (3)	C11—C12	1.438 (5)
Zn1—N3	2.067 (3)	C12—C13	1.340 (6)
Zn1—N2	2.211 (3)	C13—H13	0.9500
Zn1—N4	2.159 (4)	C6—H6A	0.9900
Zn1—N1	2.082 (3)	C6—H6B	0.9900
Cl1—O5	1.445 (3)	C6—C7	1.520 (6)
Cl1—O3	1.433 (3)	C7—H7A	0.9900
Cl1—O6	1.429 (3)	C7—H7B	0.9900
Cl1—O4	1.429 (4)	C3—H3A	0.9900
F1—C12	1.348 (5)	C3—H3B	0.9900
O2—C14	1.255 (5)	C3—C4	1.517 (7)
O1—C11	1.240 (5)	C5—H5A	0.9900
N5—C11	1.381 (5)	C5—H5B	0.9900
N5—C14	1.365 (5)	C5—C4	1.513 (7)
N6—H6	0.8800	C2—H2A	0.9900
N6—C14	1.363 (5)	C2—H2B	0.9900
N6—C13	1.377 (5)	C2—C1	1.517 (7)
N3—H3	1.0000	C8—H8A	0.9900
N3—C6	1.489 (5)	C8—H8B	0.9900
N3—C5	1.486 (5)	C8—C9	1.516 (7)
N2—H2	1.0000	C1—H1A	0.9900
N2—C3	1.476 (5)	C1—H1B	0.9900
N2—C2	1.462 (6)	C4—H4A	0.9900
N4—H4	1.0000	C4—H4B	0.9900
N4—C7	1.476 (6)	C10—H10A	0.9900
N4—C8	1.478 (6)	C10—H10B	0.9900
N1—H1	1.0000	C10—C9	1.501 (7)
N1—C1	1.479 (6)	C9—H9A	0.9900
N1—C10	1.469 (6)	C9—H9B	0.9900
			
N5—Zn1—N3	115.88 (13)	N3—C6—H6B	109.8
N5—Zn1—N2	90.91 (13)	N3—C6—C7	109.2 (3)
N5—Zn1—N4	100.68 (13)	H6A—C6—H6B	108.3
N5—Zn1—N1	108.17 (13)	C7—C6—H6A	109.8
N3—Zn1—N2	91.50 (13)	C7—C6—H6B	109.8
N3—Zn1—N4	83.40 (14)	N4—C7—C6	109.8 (3)
N3—Zn1—N1	135.54 (13)	N4—C7—H7A	109.7
N4—Zn1—N2	168.41 (14)	N4—C7—H7B	109.7
N1—Zn1—N2	81.80 (14)	C6—C7—H7A	109.7
N1—Zn1—N4	94.55 (15)	C6—C7—H7B	109.7
O3—Cl1—O5	109.9 (2)	H7A—C7—H7B	108.2
O6—Cl1—O5	108.3 (2)	N2—C3—H3A	109.2
O6—Cl1—O3	109.1 (2)	N2—C3—H3B	109.2
O6—Cl1—O4	109.9 (2)	N2—C3—C4	112.2 (3)
O4—Cl1—O5	109.6 (2)	H3A—C3—H3B	107.9
O4—Cl1—O3	110.0 (2)	C4—C3—H3A	109.2
C11—N5—Zn1	119.6 (2)	C4—C3—H3B	109.2
C14—N5—Zn1	117.8 (2)	N3—C5—H5A	109.1
C14—N5—C11	122.5 (3)	N3—C5—H5B	109.1
C14—N6—H6	119.2	N3—C5—C4	112.6 (3)
C14—N6—C13	121.6 (3)	H5A—C5—H5B	107.8
C13—N6—H6	119.2	C4—C5—H5A	109.1
Zn1—N3—H3	105.9	C4—C5—H5B	109.1
C6—N3—Zn1	109.8 (2)	N2—C2—H2A	109.8
C6—N3—H3	105.9	N2—C2—H2B	109.8
C5—N3—Zn1	117.9 (2)	N2—C2—C1	109.2 (3)
C5—N3—H3	105.9	H2A—C2—H2B	108.3
C5—N3—C6	110.5 (3)	C1—C2—H2A	109.8
Zn1—N2—H2	106.6	C1—C2—H2B	109.8
C3—N2—Zn1	119.1 (3)	N4—C8—H8A	108.9
C3—N2—H2	106.6	N4—C8—H8B	108.9
C2—N2—Zn1	104.2 (3)	N4—C8—C9	113.5 (4)
C2—N2—H2	106.6	H8A—C8—H8B	107.7
C2—N2—C3	112.9 (3)	C9—C8—H8A	108.9
Zn1—N4—H4	107.9	C9—C8—H8B	108.9
C7—N4—Zn1	104.5 (3)	N1—C1—C2	109.6 (3)
C7—N4—H4	107.9	N1—C1—H1A	109.7
C7—N4—C8	113.3 (3)	N1—C1—H1B	109.7
C8—N4—Zn1	115.0 (3)	C2—C1—H1A	109.7
C8—N4—H4	107.9	C2—C1—H1B	109.7
Zn1—N1—H1	105.2	H1A—C1—H1B	108.2
C1—N1—Zn1	110.4 (3)	C3—C4—H4A	108.5
C1—N1—H1	105.2	C3—C4—H4B	108.5
C10—N1—Zn1	117.4 (3)	C5—C4—C3	115.0 (4)
C10—N1—H1	105.2	C5—C4—H4A	108.5
C10—N1—C1	112.2 (3)	C5—C4—H4B	108.5
O1—C11—N5	120.7 (3)	H4A—C4—H4B	107.5
O1—C11—C12	123.6 (4)	N1—C10—H10A	108.9
N5—C11—C12	115.7 (4)	N1—C10—H10B	108.9
O2—C14—N5	120.5 (3)	N1—C10—C9	113.5 (4)
O2—C14—N6	120.3 (3)	H10A—C10—H10B	107.7
N6—C14—N5	119.1 (3)	C9—C10—H10A	108.9
F1—C12—C11	117.7 (4)	C9—C10—H10B	108.9
C13—C12—F1	120.2 (4)	C8—C9—H9A	108.6
C13—C12—C11	122.0 (4)	C8—C9—H9B	108.6
N6—C13—H13	120.5	C10—C9—C8	114.8 (4)
C12—C13—N6	119.0 (4)	C10—C9—H9A	108.6
C12—C13—H13	120.5	C10—C9—H9B	108.6
N3—C6—H6A	109.8	H9A—C9—H9B	107.6
			
Zn1—N5—C11—O1	−1.8 (5)	N2—C2—C1—N1	−55.9 (4)
Zn1—N5—C11—C12	176.4 (3)	N4—C8—C9—C10	−73.7 (5)
Zn1—N5—C14—O2	2.3 (5)	N1—C10—C9—C8	73.2 (5)
Zn1—N5—C14—N6	−177.4 (2)	C11—N5—C14—O2	178.8 (3)
Zn1—N3—C6—C7	33.7 (4)	C11—N5—C14—N6	−1.0 (5)
Zn1—N3—C5—C4	−59.2 (4)	C11—C12—C13—N6	1.7 (6)
Zn1—N2—C3—C4	48.6 (4)	C14—N5—C11—O1	−178.2 (3)
Zn1—N2—C2—C1	46.3 (4)	C14—N5—C11—C12	0.0 (5)
Zn1—N4—C7—C6	44.9 (4)	C14—N6—C13—C12	−2.8 (6)
Zn1—N4—C8—C9	52.6 (4)	C13—N6—C14—O2	−177.4 (3)
Zn1—N1—C1—C2	34.4 (4)	C13—N6—C14—N5	2.4 (5)
Zn1—N1—C10—C9	−53.3 (4)	C6—N3—C5—C4	173.4 (3)
F1—C12—C13—N6	−180.0 (3)	C7—N4—C8—C9	172.6 (4)
O1—C11—C12—F1	−0.6 (6)	C3—N2—C2—C1	−84.4 (4)
O1—C11—C12—C13	177.7 (4)	C5—N3—C6—C7	165.5 (3)
N5—C11—C12—F1	−178.7 (3)	C2—N2—C3—C4	171.2 (4)
N5—C11—C12—C13	−0.4 (6)	C8—N4—C7—C6	−80.9 (4)
N3—C6—C7—N4	−54.1 (4)	C1—N1—C10—C9	177.2 (4)
N3—C5—C4—C3	75.5 (4)	C10—N1—C1—C2	167.4 (3)
N2—C3—C4—C5	−69.3 (5)		

### Hydrogen-bond geometry (Å, º)

**Table:**

*D*—H···*A*	*D*—H	H···*A*	*D*···*A*	*D*—H···*A*
N6—H6···O2^i^	0.88	1.94	2.803 (4)	167
N1—H1···O1	1.00	2.19	2.966 (4)	133
N3—H3···O2	1.00	2.37	3.066 (4)	126
N3—H3···O5	1.00	2.24	3.055 (4)	138
N4—H4···O5^ii^	1.00	2.44	3.403 (5)	161
N4—H4···O6^ii^	1.00	2.44	3.229 (5)	136

Symmetry codes: (i) −*x*+1, −*y*+1, −*z*+1; (ii) −*x*+1, *y*−1/2, −*z*+3/2.
